# MRI findings of radiation-induced changes of masticatory muscles: a systematic review

**DOI:** 10.1186/1916-0216-42-26

**Published:** 2013-03-28

**Authors:** Mohammed AQ Al-Saleh, Jacob L Jaremko, Humam Saltaji, John Wolfaardt, Paul W Major

**Affiliations:** 1 Orthodontic Graduate Program, School of Dentistry, 476 Edmonton Clinic Health Academy (ECHA), University of Alberta, Edmonton, AB T6G 1C9, Canada; 2 Department of Radiology and Diagnostic Imaging, Faculty of Medicine and Dentistry, University of Alberta, 2A2.41 WC Mackenzie Health Science Center, Edmonton, AB T6G 2R7, Canada; 3 Canada Edmonton Clinic Health Academy (ECHA), Edmonton, AB, Canada; 4 Division of Otolaryngology Head and Neck Surgery, Department of Surgery, Faculty of Medicine and Dentistry, University of Alberta/Director of Clinics and International Relations, Institute of Reconstructive Sciences in Medicine, Misericordia Community Hospital, 16940-87 Avenue, Edmonton, AB T5R 4H5, Canada; 5 Faculty of Medicine and Dentistry, 5-748, University of Alberta, Edmonton, AB T6G 1C9, Canada

**Keywords:** MRI, TMJ, Masticatory muscles, Evidence-based, Radiotherapy

## Abstract

**Background:**

Radiotherapy to the head and neck regions can result in serious consequences to the temporomandibular joint (TMJ) and chewing muscles. Magnetic resonance imaging (MRI) demonstrates soft-tissue alterations after radiotherapy, such as morphology and signal intensity.

**Objective:**

The purpose of this review is to critically and systematically analyse the available evidence regarding the masticatory muscles alterations, as demonstrated on MRI, after radiotherapy for head and neck cancer.

**Data sources:**

Electronic search of MEDLINE, EMBASE, EBM reviews and Scopus.

**Inclusion criteria:**

Reports of any study design investigating radiation-induced changes in masticatory muscles after radiotherapy in patients with head and neck cancer were included.

**Results and synthesis methods:**

An electronic database search resulted in 162 papers. Sixteen papers were initially selected as potentially relevant studies; however, only four papers satisfied all inclusion criteria. The included papers focused on the MRI appearance of masticatory muscles following radiotherapy protocol. Two papers reported outcome based on retrospective clinical and imaging records, whereas the remaining two papers were case reports. Irradiated muscles frequently show diffuse increase in T2 signal and post-gadolinium enhancement post-irradiation. Also, muscle size changes were reported based on subjective comparison with the contralateral side. The quality of all included papers was considered poor with high risk of bias.

**Conclusion:**

There is no evidence that MRI interpretations indicate specific radiation-induced changes in masticatory muscles. There is a clear need for a cohort study comparing patients with pre- and post-radiotherapy MRI.

## Introduction

Radiotherapy to the head and neck regions can result in serious consequences to the temporomandibular joint (TMJ) and surrounding muscular structures. Ionizing radiation leads to tissue changes that appear as trismus, mucositis, loss of salivary gland function, osteoradionecrosis and consequent dental decay, as well as potential systemic complications [1-3]. Furthermore, radiation-induced masticatory muscle fibrosis, inflammation and atrophy reduce jaw mobility and induce trismus [3]. The severity of these effects can be related to the radiation dose received and the amount of tissues involved [4]. It has been suggested that as the radiation dose to the TMJ and pterygoid muscles is increased, the maximal jaw opening decreased linearly [4]. Trismus appears to be a consequence of irradiation of pterygoid muscles, since no similar effects were observed with irradiation of the TMJ only [5]. Radiation-induced trismus remains poorly understood. In addition, high-dose radiation of the TMJ and pre-auricular area increases the incidence of mandibular osteoradionecrosis up to 20% [5].

Magnetic resonance imaging (MRI) demonstrates soft tissue alterations after radiotherapy, such as changes in muscle morphology and signal intensity [6-9]. The radiation-induced changes to muscle that can be detected using MRI include fibrosis, inflammation, and denervation atrophy [7,8,10]. However, there are few published studies assessing these changes.

The early and late radiation-induced changes of the oral tissues can explain the associated clinical symptoms, damages to the oral mucosa, taste, salivary glands, alveolar bone, dentition, periodontium, masticatory muscles and TMJ [3]. The TMJ and the masticatory muscles are radiation late-responding tissues compared to the other oral tissues. Due to their late-response, the radiation-induced changes of TMJ and masticatory muscles may not become evident for months or years, and are often confused with tumor recurrence at the primary tumor location [3,11,12].

The five-year survival rate of the oral & oropharyngeal cancer patients is 80% for early cancer stages and 35% for late cancer stages. The main goal of cancer therapy, after survival, is to maintain normal oral functions and reduce injury to the best possible level. Understanding the mechanisms contributing to the damages including masticatory muscles, TMJ, nerve injury and trismus is the key to prevent oral functions limitation and quality of life deterioration. The late-response of TMJ and masticatory muscles and the possible confusion of tumor recurrence, emphasize the importance to distinguish the tissue alterations using an objective diagnostic tool such as MRI. Such early detection of tissue alterations ensures timely management of clinical symptoms.

The purpose of this review is to critically and systematically analyse the available evidence regarding the TMJ and masticatory muscles alterations, as demonstrated on MRI, after radiotherapy for head and neck cancer.

## Materials & methods

### Search strategy

Databases including MEDLINE (1948 to 2013 Week 1), EMBASE (1980 to 2013 Week 1), All EBM Reviews-Cochrane DSR, ACP Journal Club, Health Technology Assessment, NHS Economic Evaluation (1980 through 4th Quarter 2012), and Scopus (1965 through January 6, 2013) were systematically searched in all languages. Keywords used in the search were *Magnetic resonance imaging, imaging, MRI, MR, enhancement, signal alternations, masticatory muscles, neoplasms, cancer, carcinoma, head and neck, oral cavity, oropharynx, nasopharynx, radiotherapy, radiation therapy, intensity modulated radiotherapy, and medical radiation.* Keywords were also searched in a selection of both truncated (multiple parameter) and MeSH terms, with the help of a librarian who specializes in health sciences databases. The literature search was complemented by a manual search of the bibliographies of the identified papers (Additional file 1).

### Criteria for considering papers for this review

#### Type of study design

We included reports of any study design (clinical trials, cohort, case–control, cross-sectional studies, case reports and case series, whether prospective or retrospective) investigating radiation-induced changes of masticatory muscles after radiotherapy in patients with head and neck cancer. Editorials, anecdotal letters, and literature reviews were excluded.

#### Type of participants

Studies were on humans of any age or sex, as long as they had head and neck cancer treated with radiotherapy.

#### Type of outcome measures

Any MRI features represent changes of the muscles of mastication; whether anatomical (e.g. length, cross-section and volume of muscles) or physiological (e.g. signal intensity or contrast enhancement) were included.

### Method for considering papers for this review

Two independent reviewers (M.A. & H.S.) thoroughly screened the titles and abstracts that appeared in the database search. The reviewers selected papers, which appeared definitely or potentially relevant for further evaluation. The full text of these papers was analysed by the same reviewers independently according to the inclusion criteria (yes/no). Papers in which there was doubt as to whether inclusion criteria were met underwent re-evaluation by the reviewers to reach a decision.

### Quality assessment of the reviewed studies

We critically appraised each included study to determine the methodological quality and risk of bias, using a design-specific criteria tool intended to evaluate retrospective studies and case series [13]. The items evaluated by this tool included the study design and subject recruitment (selection bias), methods to evaluate the intervention and outcome (detection/measurement bias), statistical analysis and interpretation (analysis/interpretation bias), and effects of adjuvant intervention (performance bias). Two readers (M.A. and H.S.) were trained to use this evaluation tool on five studies of topics unrelated to this review, followed by a pilot test using another five studies. Issues and conflicts were identified from both processes of training and pilot testing. The final customized evaluation tool was then revised to reach consensus. The two reviewers (M.A. and H.S.) then independently completed the evaluation of each paper, and the results were compared. At this stage, agreement between the reviewers for grading on papers was calculated using intra-class correlation coefficients (ICC).

## Results

**Figure 1:**
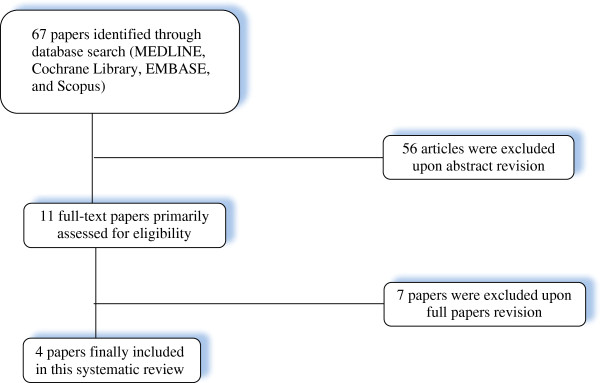
Flow diagram of the selection process.

The initial database search yielded 67 papers. Primary review of titles and abstracts narrowed this to 11 potentially relevant studies. Based on full-text review, only four of these papers met all inclusion criteria (Figure 1) [14-17].

### Characteristics of included papers

Information on the study patient’s *demographics, study design, tumor and treatment types, method and time of data collection, clinical findings, MRI findings and radiographic findings* of the included papers are outlined in Table 1.

**Table 1:** Sample demographics, study design, tumor type, treatment type, method and timing of data collection of the selected papers

	**Pajari et al., 1996 **[17]	**Chong et al. 2000 **[16]	**Ariji et al. 2002 **[14]	**Bhatia et al. 2009 **[15]
**Study design**	Case report	Retrospective study	Case Report	Retrospective study (1998–2005)
**How information was obtained**	• Clinical and MRI assessment.	• Review of clinical and MRI records.	• Clinical and MRI assessment.	• Review of clinical and MRI records.
**Population**	• N = 1	• N = 5 (only 3 had MRI)	• N = 1	• N = 35
**Characteristics**	• Sex: F	• Sex: 4 M, 1 F.	• Sex: M	• Sex: 30 M, 5 F.
**Mean age**	• Age: 9 years.	• Age: mean 54 years, range 17 to 74.	• Age: 57 years.	• Age: mean 51 years, range 35 to 75.
**Tumor type**	• Rhabdomyosarcoma	• Squamous cell carcinoma.	• Squamous cell carcinoma.	• Nasopharyngeal carcinoma
		• Adenocarcinoma.		
		• Mucoepidermoid and oncocytic carcinomas of parotid.		
**Treatment type**	• Radiotherapy	• Radiotherapy	• Radiotherapy	• Radiotherapy
	• Chemotherapy		• Chemotherapy	
**Tumor site**	Right auricular region	Soft palate	Nasopharynx	Nasopharynx
		Nasopharynx		
		Submandibular and parotid glands		
**Time between radiotherapy completion and MRI assessment**	• 18 months	• Mean 4.4 years (range 1 to 8 years)	• 8 months	• Mean 6.7 years (range 1.3 to 15.2 years)
**Radiation dose**	• 50 Gy	• 60 to 69 Gy	• 75.8 Gy	• Not reported
**MRI findings**	○ (T1-W):	○ (T1-W, T2-W, contrast-enhanced):	○ (T1-W, T2-W, contrast-enhanced):	○ (T1-W, T2-W, contrast-enhanced):
			***Pre-radiotherapy:***	
	• Ipsilateral masseter muscle atrophy.	• Bone marrow of involved portion of mandible showed homogenous low signal intensity in T1-W, high signal intensity in T2-W, and diffuse intense enhancement with contrast medium.	• Tumor mass in left nasal cavity.	• 19 patients had abnormal increase in signal intensity of masseter, temporalis, lateral & medial pterygoid muscles
	• Ipsilateral condylar head flattening.	• All patients had cortical disruption of the mandible.	• Ipsilateral size reduction in the masseter, lateral & medial pterygoid muscles.	.• 16 patients had only mild signal intensity changes in masticatory muscles. However, they had different abnormalities such as (nerve injury, TMJ deformity, osteoradionecrosis, fibrosis & inflammation).
	• Contralateral condylar head osteophyte.	• 3 patients had ipsilateral increase of signal intensity of masseter, lateral and medial pterygoid muscles adjacent to the osseous abnormalities.	• T1-W revealed increase in signal intensity of the lateral pterygoid muscle due to fatty infiltration.	
	• Bilateral normal TMJ articular disc morphology.	• 2 patients had prominent mass-like thickening of masseter, lateral and medial pterygoid muscles.	• T2-W revealed increase of signal intensity of the masseter, lateral & medial pterygoid, temporalis and mylohyoid muscles.	
	• Bilateral normal signal intensity of the TMJ articular surfaces & mandibular ramus.		• Contrast enhanced image showed tumor invasion along the mandibular division of trigeminal nerve.	
			***Post- radiotherapy:***	
			• Remarkable increase in the (T2-W) signal intensity of the lateral pterygoid muscle.	
**Radiographic findings**	***○ Panoramic radiograph:***	***○ CT with IV administration of contrast medium:***	-----------	------------
	• Normal TMJ articular disc morphology.	• All patients had ipsilateral abnormal enhancement of the masseter, lateral and medial pterygoid muscles adjacent to the osseous abnormalities.		
	• Root development stopped at ipsilateral molars and premolars.	• 4 patients had prominent mass-like thickening of masseter, lateral and medial pterygoid muscles.		
	• Ipsilateral shorter ramus and larger gonial angle.	• All patients had mandibular osseous abnormality, disorganization & loss of trabeculation of the spongiosa of the mandible.		
		• 1 patient suffered ipsilateral mandibular fistula.		
		• 1 patient suffered ipsilateral mandibular pathologic fracture.		
**Clinical findings**	• Tenderness of ipsilateral TMJ capsule, masseter, lateral & medial pterygoid and posterior digastric muscles.	• All patient suffered ipsilateral facial pain and swilling.	-----------	• Restricted vertical mouth opening range (3–25 mm).
	• Restricted vertical mouth opening (23mm).	• 1 patient suffered ipsilateral numbness, tingling & dysesthesia a long the inferior alveolar nerve.		
	• Normal lateral mouth movement.	• 3 patients suffered trismus.		
	• Higher EMG activity of ipsilateral masseter & temporalis muscles in all movements.	• All patients suffered osteoradionecrosis (4 ipsilateral &1 contralateral).		
	• Normal salivary flow.	• 1 patient suffered ipsilateral mandibular fistula.		
	• Ipsilateral superficial dental decay, heavy plaque accumulation and gingivitis.	• 1 patient suffered ipsilateral mandibular pathologic fracture.		

The four included papers focused on the MRI appearance of the masticatory muscles following different radiotherapy protocol [14-17]. Tumors varied, with histological types reported in three papers [14,16,17]. Two papers reported outcome based on retrospective clinical and imaging assessment records [15,16], whereas the remaining two papers were case reports [14,17]. In the first included study, Chong et al. retrospectively screened medical records of five patients who reported facial pain, mandibular osteoradionecrosis and reduced range of motion (trismus) at different follow-up times after radiotherapy [16]. Only three patients had MRI available, 1–8 years post radiotherapy completion. On the MRI, the masseter and pterygoid muscles close to the tumor-involved portions of the mandible showed abnormally increased T2 signal intensity in the masseter and pterygoid muscles and diffuse intense enhancement post-gadolinium injection. Thickening and increase of muscle size on the radiated side was demonstrated on contrast-enhanced coronal CT scan.

In the second included study, Bhatia et al. retrospectively screened the medical records of 35 patients who had limited mandibular movement (trismus) at different follow-up times (1 to 15 years) after radiotherapy completion. MRI of temporalis, masseter and pterygoid muscles revealed substantially increased T2 signal intensity and focal post-gadolinium enhancement in at least one muscle at the irradiated side in 19 patients (59%) [15]. Atrophy of all muscles at the irradiated side (implying mandibular nerve injury) was noticed in one patient only (3%).

In the two included case reports, Pajari et al. evaluated mandibular range of motion, masticatory muscle morphology and tenderness, dental and salivary flow conditions at 18 months after radiotherapy and chemotherapy completion for a 9-year-old child [17]. On the MRI, the masseter muscle was seen on the coronal T1-W sequence to be reduced in size at the irradiated side [17]. Ariji et al. evaluated the temporalis, masseter and pterygoid muscles prior to and at 12 months after radiotherapy and chemotherapy completion for a 57-year-old male [14]. All masticatory muscles on the irradiated side showed new post-gadolinium enhancement and increased T2 signal at axial T1-W and T2-W MR imaging, respectively, implying denervation atrophy.

### Quality assessment

The four included papers were scored according to the assessment tool [14-17]. The result of the ICC between the two reviewers was 93.5% [59%-99%], which indicates excellent agreement [18]. Papers were rated as poor quality with a high risk of bias. Table 2 shows the quality assessment scores. Reviewed papers showed evidence of multiple sources of bias, such as selection, detection/measurement, statistical analysis and interpretation bias. In addition, reviewed papers did not conduct an adequate statistical analysis, and they were mainly descriptive [14-17].

**Table 2:** Quality and risk of bias assessment tool

**Parameters of evaluations**			**Pajari et al. 1996 **[17]	**Chong et al. 2000 **[16]	**Ariji et al. 2002 **[14]	**Bhatia et al. 2009 **[15]
A. Selection bias	1.	Randomized sample	N/A	No	N/A	No
	2.	Sample size ≥ 30	N/A	No	N/A	Yes
	3.	Adequate test group:	N/A	Yes	N/A	Yes
		• Were cases selected appropriately (e.g., appropriate diagnostic criteria or definitions)				
	4.	Adequate control (before and after)	No	No	Yes	No
		• Inadequate: contralateral side of same patient				
	5.	Inclusion/exclusion criteria for recruitment	N/A	No	N/A	Yes
B. Detection or measurement bias	6.	Adequate follow-up	Yes	No	No	No
		• Inadequate: or follow-up period was not the same between patients, or less than 9 months post-treatment.				
	7.	Was the intervention assessed using a reliable measure? i.e. pre-treatment muscular tissue assessment	No	No	Yes	Yes
		• Example: with MRI, CT, or others.				
	8.	Were the outcomes assessed using a reliable measure? i.e. MRI muscle tissue assessment measurements	No	No	No	No
		• Example: Inter or intra-examiner agreement reported				
	9.	Outcome assessors blinded to intervention.	No	No	No	No
	10.	Reported and statistically controlled for confounding factors.	N/A	No	N/A	No
C. Analysis or interpretation bias	11.	Adequate statistical tests used.	N/A	No	N/A	Yes
		Inadequate: e.g. univariate analysis for multivariate outcomes				
	12.	Adequate and complete reporting of results	N/A	No	N/A	No
		Inadequate: e.g. lack of SD or 95% CI, reporting significance based on *P* value when R^2^/correlation is <50%.				
D. Performance bias	13.	Did researchers rule out any impact from a concurrent intervention or an unintended exposure that might bias results?	N/A	Yes	N/A	Yes
Total score			1/5	2/13	2/5	6/13

## Discussion

Out of many papers in the literature noting clinical oral symptoms long after radiotherapy, only the four included papers have objectively evaluated the imaging manifestations of masticatory muscles and related structures directly on MRI [14-17]. This significant deficiency in the literature exists despite the potential for MRI to thoroughly evaluate these structures. MRI allows quantitative analysis of masticatory muscles, including measurement of sectional area, thickness, signal intensity, and surface characteristics [6]. This enables noninvasive identification of muscle fibrosis, inflammation, and denervation, all markers of muscle damage after radiotherapy [7,8,10]. Although radiotherapy has become essential in the treatment of head and neck cancers [19], damage to adjacent non-cancerous tissues is frequent and may appear immediately or years after irradiation [10,20]. Radiation-induced damage to vascular and neural structures and slowly proliferating connective tissue cells explains the complications and side effects that alter bone, cartilage and muscular tissues [10]. Homogeneity and size of radiation dose fractions, fraction time interval, ratio of radiation dose to the tissue volume, are factors that determine the nature and extent of the complications [10]. It is of paramount importance to distinguish muscular tissue alteration that is radiation-induced versus change due to recurrent tumor infiltration, but this can be difficult on MRI. In addition to tumor invasion, other tissue abnormalities such as pre-existing trismus or post-surgical changes can also be confused with radiation-induced symptoms. To properly evaluate radiotherapy effects on muscle, only patients with no symptoms before radiotherapy should be included in any post-radiotherapy assessment. Radiation-induced tissue injuries likely develop and heal soon after completion of radiotherapy, but residual ischemia and tissue fibrosis may not be symptomatic until months or years post radiotherapy [21-23]. A follow-up period measured in years should be considered to avoid confusion of these chronic processes with any acute or transient tissue injury immediately post radiotherapy.

### Quality assessment

Viswanathan et al. developed a design-specific criteria tool to assess risk of bias of different study designs that include retrospective studies and case series studies, based on workgroup consensus and peer review [13]. Although the authors stated that the design-specific criteria tool can be adapted to different study designs, many tool items were not applicable on the case reports we assessed. In general, case reports are weak in scientific quality and do not usually add conclusive evidence to systematic reviews. However, in this systematic review, the authors chose to consider them due to limited more-formal trials in the literature. The risk of bias of the analysed papers in this review was high, due to the lack of information regarding: tumor stage, exact location of the lesion surgical treatment applied, adequate control and adequate follow-up, control of confounding factors, blinding of outcome measures, and inter or intra-examiner reliability.

Muscle comparability and isolation of the effect of the intervention (radiation) on outcomes are important methodological factors that were missing in all included papers [14-17]. The above-mentioned methodological drawbacks yield serious concern regarding the confidence of the reported outcomes and limit the impact of their findings.

### MRI findings

All included papers reported the MRI finding of the masticatory muscles at least 12 months after radiotherapy completion [14-17].

#### Muscle signal intensity

The masticatory muscles frequently show diffuse increase in T2 signal and post-gadolinium enhancement post-irradiation. The increase in tissue water content and vascularity are nonspecific changes and can be seen as a result of several pathologic mechanisms, including radiation-induced fibrosis, residual or recurrent tumor, mandibular osteoradionecrosis, or denervation muscle atrophy [10]. Bhatia et al. reported these prominent signal abnormalities in pterygoid muscles, temporalis and/or masseter in 45% of patients who suffered post-irradiation trismus [15]. The other half of the patients showed only mildly increased T2 signal and minor enhancement, which the authors noted they believe to be a normal finding post irradiation. However, that retrospective study did not include a cohort without trismus to confirm this. In the 55% of patients without intense MRI signal abnormality it is possible that MRI could not detect functionally significant muscular fibrosis or that other disease processes may have contributed to the trismus. Chong et al. noticed that 3 of 5 patients (60%) with mandibular osteoradionecrosis also had a high T2 signal in the ipsilateral masseter and pterygoid muscles [16]. The single case of apparent denervation atrophy reported by Ariji et al. showed increased MRI signal intensity of all masticatory muscles on the irradiated side within a few months of irradiation, thought to be due to inflammation and edema-like response of muscle after acute and subacute mandibular nerve damage [14]. This case report illustrates that muscle signal changes may be complex due to combination of factors such as chronic tumor-related denervation atrophy and subacute radiation effects.

#### Muscle size

The included papers reported muscle size changes based on subjective comparison with the contralateral side. Peripheral nerves, such as the mandibular nerve, can be damaged by tumor invasion or by exposure to a very high dose of radiotherapy (more than 100 Gy) [24]. Cheng and Schultz reported that indirect radiation-induced nerve damage may occur due to extensive fibrosis of the connective tissues surrounding the nerve, and can become symptomatic a few months up to three years post-irradiation [24]. Previous radiotherapy treatment and surgical complications such as ischemia may also significantly contribute to the peripheral nerve damage [24]. Although few studies have reported cases of radiation-induced nerve damage, some authors believe that these cases are under-reported in the literature [14,24]. Alteration of the masticatory muscles due to denervation can begin with oedema, inflammation, and progress to a fatty infiltration and atrophy [7,8]. It has been reported that MRI findings of denervated masticatory muscles can be observed immediately after symptoms of paralysis are reported [7,8,25]. Russo et al. suggested specific descriptions of the MRI appearance of the masticatory muscles based on the chronicity of the nerve damage, from the onset of symptoms of paralysis up to 20 months later [8]. The authors reported that at time of paralysis onset, the affected muscle showed a high T2 signal, abnormal enhancement, muscle volume increase and no signs of fatty infiltration. A few months later, at a subacute to early chronic stage of muscle denervation, MRI showed high T2 signal intensity and abnormal enhancement, with normal muscle volume. At the long-term chronic stage, the MRI showed muscular volume loss with extensive fatty infiltration, and no high intensity signals at T2-W imaging or abnormal enhancement [8]. Ariji et al. reported a single complex case, showing the MRI appearance (T1-W and T2-W) of a patient who had masticatory muscle denervation atrophy due to cavernous sinus tumor invasion [14]. New increase in T2 signal and post-gadolinium enhancement in the atrophic masticator muscles eight months later were deemed likely to be radiotherapy-related. Bhatia et al. reported high signal intensity and denervation atrophy of the ipsilateral masticatory muscles (lateral and medial pterygoid, masseter and temporalis) in only one patient (3%) of their series, thought due to direct tumor invasion of the mandibular nerve [15]. They did not find muscle size to be a significant finding to detect radiation-induced trismus [15]. Chong et al. found that two out of three patients had prominent mass-like thickening of the masseter and pterygoid muscles adjacent to the radiation-induced osteoradionecrosis [16]. The authors did not investigate or report the possible reasons of such association between the osteoradionecrosis and the thickening of the muscular tissue. Pajari et al. reported significant muscle atrophy associated with restricted mouth opening of a 9-year-old child more than a year after radiotherapy. The authors did not emphasize any other findings regarding the muscle MRI appearance [17].

The MRI appearance of the masticatory muscles has received little attention in the literature. Distinguishing post-radiation effects from denervation atrophy and tumor recurrence requires consideration of location, clinical findings, previous images, and timing of the changes, given the expected time course of evolution of post-radiation and denervation changes. The development of muscle tissue abnormality at a distant or contralateral site to the primary tumor favours radiation-induced tissue changes.

### Recommendation for future research

Based on the risk of bias and methodological assessment, major limitations were identified. Some suggestions for future research are as follows:

1. None of the studies compared MRI findings of cohorts of irradiated patients with and without trismus. This is essential to determine which findings are specifically associated with trismus.

2. An ideal study design for clinically meaningful outcomes should include a sufficiently powered sample size, random allocation of patients and standardized intervention. Although patient random allocation and standardized radiation protocol are important to support research findings, unfortunately they are not clinically or ethically feasible in the field of cancer radiotherapy [26,27].

3. Muscle soreness was evaluated based on patients self-reporting. Valid, reliable and more objective measurement of the muscle soreness can be simply measured using algometry [28]. Trismus can be confirmed by direct measurement of mouth opening limitation and lateral movements in millimetres to avoid biased subjective assessment of mouth movement. This is important to relate MRI findings to clinical presentation.

4. There is a need to establish more objective imaging description of the muscles of mastication such as digitally measured dimensions (i.e. muscle thickness, cross-section) and muscular volume.

5. MRI scans at pre- and post-radiotherapy are necessary to establish a valid and reliable reference for the muscle tissue alterations.

## Conclusions

MRI findings of post-radiation changes in masticatory muscles in patients with head and neck cancer are nonspecific and difficult to interpret given the near-complete lack of available evidence in the literature. In particular, it is unclear which muscular changes correlate specifically to trismus, rather than simply reflecting the complex series of events in nearby tissue including tumor growth, denervation atrophy, surgery, chemotherapy and irradiation. To investigate this further, there is a clear need for a cohort study comparing patients with and without post-radiotherapy trismus, which would be particularly valuable if performed prospectively, with pre- and post-radiotherapy MRI, and with specific information regarding the radiotherapy dose and target volume.

## Competing interests

The authors declare that they have no competing interests.

## Authors’ contribution

MA carried out the electronic systematic search, screened the articles for inclusion and drafted the manuscript. HS participated in the electronic systematic search, screening for inclusion and carried out the quality assessment. JJ screened the articles for inclusion, carried out the quality assessment and drafted the manuscript. JW drafted the manuscript. PM drafted the manuscript. All authors red and approved the final manuscript.

## Supplementary Material

Additional file 1 Electronic search strategy. Click here for file
