# Supplementary material for: MRI findings of radiation-induced changes of masticatory muscles: a systematic review
Source: J Otolaryngol Head Neck Surg. 2013 Mar 28;42(1):26. doi: 10.1186/1916-0216-42-26 (PMC3651244; doi:10.1186/1916-0216-42-26)
Supplement: Additional file 1 — Electronic search strategy. [file 1916-0216-42-26-S1.docx]

- MRI Findings of Radiation-Induced Changes of Masticatory Muscles: A Systematic Review
- Electronic search strategy.

| **Database** | **Keywords** | **Results**  **After abstract screening** | **Selected** |  |
| --- | --- | --- | --- | --- |
| MEDLINE  1946 to 2013 January 04 | 1     exp Magnetic Resonance Imaging/ or MRI.mp. (316026)  2     Masticatory [muscles.mp](http://muscles.mp/). or exp Masticatory Muscles/ (11535)  3     exp Mastication/ or exp Masticatory Muscles/ or muscles of [mastication.mp](http://mastication.mp/). or exp Masseter Muscle/ or exp Temporal  Muscle/ (17482)  4     2 or 3 (17833)  5     exp Magnetic Resonance Imaging/ or MR.mp. (331673)  6     1 or 5 (362243)  7     cancer*.mp. (997498)  8     neoplasm*.mp. or exp Neoplasms/ (2468361)  9     exp Carcinoma/ or carcinoma*.mp. (690453)  10     7 or 8 or 9 (2680204)  11     (head and neck).mp. [mp=title, abstract, original title, name of substance word, subject heading word, protocol  supplementary concept, rare disease supplementary concept, unique identifier] (74720)  12     oral [cavity.mp](http://cavity.mp/). or exp Mouth/ (241414)  13     exp Mouth Neoplasms/ or exp Oropharyngeal Neoplasms/ or [oropharyngeal.mp](http://oropharyngeal.mp/). or exp Oropharynx/ (71930)  14     exp Nasopharyngeal Neoplasms/ or [nasopharyngeal.mp](http://nasopharyngeal.mp/). or exp Nasopharynx/ (26824)  15     11 or 12 or 13 or 14 (380809)  16     exp Radiotherapy/ or [radiotherapy.mp](http://radiotherapy.mp/). (190748)  17     Oncology.mp. or exp Medical Oncology/ or exp Radiation Oncology/ (55580)  18     exp Radiotherapy, Intensity-Modulated/ or radiation [therapy.mp](http://therapy.mp/). (49039)  19     16 or 17 or 18 (258218)  20     4 and 6 and 10 and 15 and 19 (9)  21     [imaging.mp](http://imaging.mp/). (610483)  22     signal [alterations.mp](http://alterations.mp/). (227)  23     [enhancement.mp](http://enhancement.mp/). or exp Image Enhancement/ (506602)  24     21 or 22 or 23 (956799)  25     6 or 24 (1015251)  26     4 and 10 and 15 and 19 and 25 (18) | 6 | 4 |  |
| EMBASE  1974 to 2013 January 04 | 1     exp Magnetic Resonance Imaging/ or MRI.mp. (483768)  2     Masticatory [muscles.mp](http://muscles.mp/). or exp Masticatory Muscles/ (7116)  3     exp Mastication/ or exp Masticatory Muscles/ or muscles of [mastication.mp](http://mastication.mp/). or exp Masseter Muscle/ or exp Temporal  Muscle/ (21743)  4     2 or 3 (22047)  5     exp Magnetic Resonance Imaging/ or MR.mp. (503121)  6     1 or 5 (522574)  7     cancer*.mp. (1972087)  8     neoplasm*.mp. or exp Neoplasms/ (3167887)  9     exp Carcinoma/ or carcinoma*.mp. (901888)  10     7 or 8 or 9 (3462009)  11     (head and neck).mp. [mp=title, abstract, subject headings, heading word, drug trade name, original title, device  manufacturer, drug manufacturer, device trade name, keyword] (99936)  12     oral [cavity.mp](http://cavity.mp/). or exp Mouth/ (174332)  13     exp Mouth Neoplasms/ or exp Oropharyngeal Neoplasms/ or [oropharyngeal.mp](http://oropharyngeal.mp/). or exp Oropharynx/ (98097)  14     exp Nasopharyngeal Neoplasms/ or [nasopharyngeal.mp](http://nasopharyngeal.mp/). or exp Nasopharynx/ (33008)  15     11 or 12 or 13 or 14 (343084)  16     exp Radiotherapy/ or [radiotherapy.mp](http://radiotherapy.mp/). (400584)  17     Oncology.mp. or exp Medical Oncology/ or exp Radiation Oncology/ (140236)  18     exp Radiotherapy, Intensity-Modulated/ or radiation [therapy.mp](http://therapy.mp/). (72034)  19     16 or 17 or 18 (523963)  20     4 and 6 and 10 and 15 and 19 (34)  21     [imaging.mp](http://imaging.mp/). (857606)  22     signal [alterations.mp](http://alterations.mp/). (280)  23     [enhancement.mp](http://enhancement.mp/). or exp Image Enhancement/ (223316)  24     21 or 22 or 23 (1013770)  25     6 or 24 (1066093)  26     4 and 10 and 15 and 19 and 25 (48) | 4 | 1 |  |
| EBM Reviews - Cochrane Database of Systematic Reviews <2005 to November 2012>, EBM Reviews - ACP Journal Club  <1991 to December 2012>, EBM Reviews - Database of Abstracts of Reviews of Effects <4th Quarter 2012>, EBM Reviews -  Cochrane Central Register of Controlled Trials <December 2012>, EBM Reviews - Cochrane Methodology Register <3rd Quarter  2012>, EBM Reviews - Health Technology Assessment <4th Quarter 2012>, EBM Reviews - NHS Economic Evaluation Database  <4th Quarter 2012> | 1     exp Magnetic Resonance Imaging/ or MRI.mp. (5838)  2     Masticatory [muscles.mp](http://muscles.mp/). or exp Masticatory Muscles/ (308)  3     exp Mastication/ or exp Masticatory Muscles/ or muscles of [mastication.mp](http://mastication.mp/). or exp Masseter Muscle/ or exp Temporal  Muscle/ (462)  4     2 or 3 (510)  5     exp Magnetic Resonance Imaging/ or MR.mp. (5672)  6     1 or 5 (7147)  7     cancer*.mp. (50385)  8     neoplasm*.mp. or exp Neoplasms/ (45926)  9     exp Carcinoma/ or carcinoma*.mp. (18176)  10     7 or 8 or 9 (72130)  11     (head and neck).mp. [mp=ti, ab, tx, kw, ct, ot, sh, hw] (3919)  12     oral [cavity.mp](http://cavity.mp/). or exp Mouth/ (6743)  13     exp Mouth Neoplasms/ or exp Oropharyngeal Neoplasms/ or [oropharyngeal.mp](http://oropharyngeal.mp/). or exp Oropharynx/ (1431)  14     exp Nasopharyngeal Neoplasms/ or [nasopharyngeal.mp](http://nasopharyngeal.mp/). or exp Nasopharynx/ (1109)  15     11 or 12 or 13 or 14 (12387)  16     exp Radiotherapy/ or [radiotherapy.mp](http://radiotherapy.mp/). (11152)  17     Oncology.mp. or exp Medical Oncology/ or exp Radiation Oncology/ (9056)  18     exp Radiotherapy, Intensity-Modulated/ or radiation [therapy.mp](http://therapy.mp/). (3000)  19     16 or 17 or 18 (19828)  20     4 and 6 and 10 and 15 and 19 (0)  21     [imaging.mp](http://imaging.mp/). (11833)  22     signal [alterations.mp](http://alterations.mp/). (1)  23     [enhancement.mp](http://enhancement.mp/). or exp Image Enhancement/ (9089)  24     21 or 22 or 23 (18480)  25     6 or 24 (20245)  26     4 and 10 and 15 and 19 and 25 (0) | 0 | 0 |  |
| Scopus  1965 to January 6, 2013 | 1. TITLE-ABS-KEY(**magnetic** **resonance** **imaging** OR **mri** OR **mr**) (484,334)  TITLE-ABS-KEY(**imaging**) (1.159,159)  TITLE-ABS-KEY(**signal** **alteration**) (28,013)  TITLE-ABS-KEY(**enhancement**) (476,951)  2. TITLE-ABS-KEY(**cancer** OR **neoplasm** OR **carcinoma**) (2,739,402)  3. TITLE-ABS-KEY(**"Head and Neck"** OR **oropharynx** OR **nasopharynx** OR **"oral cavity"**) (137,358)  4. TITLE-ABS-KEY(**radiotherapy** OR **"radiation therapy"**) (281,722)  5. TITLE-ABS-KEY(**"masticatory muscles"** OR **muscles**) (1,173,433)  6. (TITLE-ABS-KEY(**cancer** OR **carcinoma** OR **neoplasm**)) AND (TITLE-ABS-KEY(**"Head and Neck"** OR **oropharynx** OR **nasopharynx** OR **"oral cavity"**)) AND (TITLE-ABS-KEY(**"masticatory muscles"** OR **muscles**)) AND ((TITLE-ABS-KEY(**magnetic** **resonance** **imaging** OR **mri** OR **mr**)) AND (TITLE-ABS-KEY(**imaging**)) AND (TITLE-ABS-KEY(**signal** **alteration**)) AND (TITLE-ABS-KEY(**enhancement**))) AND (TITLE-ABS-KEY(**radiotherapy** OR **"radiation therapy"**)) (1) | 1 | 1 |  |
| Subtotal |  |  | 6 |  |
| Repeated |  |  | 2 |  |
| Total to be analyze for inclusion |  | 11 | 4 |  |
| Manual search to be analyze for inclusion |  | 00 | 00 |  |
| Total |  |  | 4 |  |
